# Intensity-modulated radiotherapy of nasopharyngeal carcinoma: a comparative treatment planning study of photons and protons

**DOI:** 10.1186/1748-717X-3-4

**Published:** 2008-01-24

**Authors:** Zahra Taheri-Kadkhoda, Thomas Björk-Eriksson, Simeon Nill, Jan J Wilkens, Uwe Oelfke, Karl-Axel Johansson, Peter E Huber, Marc W Münter

**Affiliations:** 1 Göteborg University and Department of Oncology, Sahlgrenska University Hospital, Göteborg, Sweden; 2 Department of Medical Physics in Radiation Oncology, German Cancer Research Center (DKFZ), Heidelberg, Germany; 3 Department of Radiophysics, Sahlgrenska University Hospital, Göteborg, Sweden; 4 Clinical Cooperation Unit Radiation Oncology, German Cancer Research Center (DKFZ), Heidelberg, Germany

## Abstract

**Background:**

The aim of this treatment planning study was to investigate the potential advantages of intensity-modulated (IM) proton therapy (IMPT) compared with IM photon therapy (IMRT) in nasopharyngeal carcinoma (NPC).

**Methods:**

Eight NPC patients were chosen. The dose prescriptions in cobalt Gray equivalent (Gy_E_) for gross tumor volumes of the primary tumor (GTV-T), planning target volumes of GTV-T and metastatic (PTV-TN) and elective (PTV-N) lymph node stations were 72.6 Gy_E_, 66 Gy_E_, and 52.8 Gy_E_, respectively. For each patient, nine coplanar fields IMRT with step-and-shoot technique and 3D spot-scanned three coplanar fields IMPT plans were prepared. Both modalities were planned in 33 fractions to be delivered with a simultaneous integrated boost technique. All plans were prepared and optimized by using the research version of the inverse treatment planning system KonRad (DKFZ, Heidelberg).

**Results:**

Both treatment techniques were equal in terms of averaged mean dose to target volumes. IMPT plans significantly improved the tumor coverage and conformation (*P *< 0.05) and they reduced the averaged mean dose to several organs at risk (OARs) by a factor of 2–3. The low-to-medium dose volumes (0.33–13.2 Gy_E_) were more than doubled by IMRT plans.

**Conclusion:**

In radiotherapy of NPC patients, three-field IMPT has greater potential than nine-field IMRT with respect to tumor coverage and reduction of the integral dose to OARs and non-specific normal tissues. The practicality of IMPT in NPC deserves further exploration when this technique becomes available on wider clinical scale.

## Background

Radiotherapy (RT) of nasopharyngeal carcinoma (NPC) is a challenging task. While distant dissemination is the most common site of failure, local recurrence occurs still in more than one-third of patients with locally advanced disease (T3–T4) treated with two-dimensional RT (2D-RT) only [1]. Furthermore, the nasopharyngeal cavity is surrounded by critical neural tissues and sensitive structures such as auditory apparatus, temporomandibular (TM) joints, and parotid glands whose normal functioning is essential for maintenance of the patients' overall well-being. A quality of life (QoL) study of patients with head and neck cancer by Huguenin et al. [2] revealed that NPC patients had the highest morbidity probably as the result of using large RT fields which included the salivary glands and TM joints. In another QoL survey of disease-free NPC patients, xerostomia, hearing impairment, dysphagia and trismus were reported as the most frequent side effects when RT was delivered by conventional techniques [3,4]. Since implementation of three-dimensional conformal RT (3D-CRT), clear definition of target volumes and organs at risk (OARs) and accurate estimation of tissue heterogeneities have become available which may account for the 3-year local control rate above 80% for T3–T4 tumors reported in some studies [5]. Nevertheless, simultaneous protection of several OARs and optimization of dose homogeneity and conformity to the concave and often irregularly-shaped target volumes in NPC have been beyond the operational scope of 3D-CRT. In recent years, intensity-modulated RT using photons (IMRT) have been applied clinically for NPC patients for whom the dosimetric advantges of this technique have contributed to improving tumor-free survival rates and reducing RT-related side effects such as xerostomia [6,7]. However, for T3–T4 tumors, a 3-year local failure rate of 17% is reported despite using whole course IMRT [7]. Interestingly, while evaluation of QoL scores (EORTC QLQ-C30 and EORTC QLQ-HN35) in NPC patients has revealed the superiority of 3D-CRT or IMRT over 2D-RT +/- 3D-CRT techniques, it could not show any significant difference between 3D-CRT and IMRT [8].

Recently, much interest is devoted to application of protons in the treatment of head and neck cancers [9-11]. The dosimetric characteristics of protons, with sharp distal fall-off of the dose in combination with technical improvements in treatment planning and dose delivery using intensity modulation (IMPT) and 3D spot-scanning [12-14] can lead to more conformal dose distributions of protons in vivo. The advantages of IMPT over state-of-the-art IMRT in the head and neck region have been demonstrated by comparative planning studies [15,16] revealing dosimetric benefits, essentially by lowering the integral dose in OARs and non-critical normal tissues.

In this paper, we present a simulation study which investigates the potential benefits of IMPT over IMRT in the treatment of NPC patients with regard to target volumes, OARs and non-specific normal tissues. Since this project is a simulation work, the predictive effects of tumour histology or chemotherapy were not taken into consideration.

## Methods

### Patient selection and target/OAR definition

Eight patients including two pediatric cases, with a histologically proven diagnosis of NPC were selected. These patients were being treated at the Department of Radiotherapy, Sahlgrenska University Hospital, Göteborg, Sweden. Their TNM stages according to the 1997 American Joint Committee on Cancer staging system were: T_1_N_0_M_0_; T_1_N_1_M_0_; T_2a_N_3a_M_0_; T_2b_N_3b_M_0_; T_3_N_2_M_0_; T_3_N_3b_M_0_; T_4_N_1_M_0_; T_4_N_2_M_0_. The original CT data sets with a slice thickness of 5–7 mm and no interslice gap were acquired and transferred to the treatment planning system, VIRTUOS, available at the German Cancer Research Center (DKFZ), Heidelberg, Germany for target definition. Based on the clinical data and pre-therapy diagnostic CT/MR images, the gross tumor volume of the primary tumor (GTV-T) and of the nodal metastases (GTV-N) were re-delineated on each CT slice. Two sets of clinical target volumes (CTV) were defined for each patient. CTV-TN was defined as the volume encompassing GTV-T and GTV-N, when present, with a 10 mm margin in all directions. The whole of the nasopharyneal cavity was also included in this volume. CTV-N consisted of the volume of the bilateral cervical lymph node stations in levels Ib to V, medial supraclavicular fossae, retro/parapharyngeal spaces, the posterior nasal cavity and maxillary sinuses, inferior sphenoidal body, clivus, and pterygoid fossae. To account for set-up errors and patient movements, two sets of planning target volumes (PTV-TN, and PTV-N) were also defined by adding a 5 mm margin to each corresponding CTV. All PTVs and CTVs were modified wherever they encountered neural tissues or bony structures without evidence of tumor infiltration. For example, for cases with T1–T2 disease or when delineating the cervical lymph node stations, only surface of the clivus and cervical vertebrae were included in PTV-TN and PTV-N, respectively. Likewise for T3–T4 tumors, in the regions where GTV-T was in close vicinity of the brainstem or optic nerves, there was no margin between GTV-T and PTV-TN meaning that the outer bounderies of both target volumes were the same in these particular regions. Since there was no clinical evidence of skin infiltration by GTV-T or GTV-N in any of the patients, PTV-TNs and PTV-Ns were always modified so that they did not extend into or out of the skin.

The mean volumes for GTV-T, PTV-TN, and PTV-N were 24.4 cc (4.3–56.1), 287.8 cc (100.9–428.7) and 450.3 cc (157.4–993.6), respectively. Besides the standard OARs (spinal cord, brainstem, temporal lobes, the optic apparatus and parotid glands), the inner and middle/external ears, cerebellum and posterior brain tissue up to the levels of the clinoids, skin, TM joints, pituitary and thyroid glands, larynx/esophagus, and the oral cavity were also delineated. All target volumes and OARs were delineated by the same radiation oncologist. The use of same treatment planning system to prepare both the IMRT and IMPT plans eliminated the risk of discrepancies for any calculated volume.

### Dose prescription and treatment planning

Dose prescriptions in cobalt Gray equivalent (Gy_E_) to GTV-T, PTV-TN and PTV-N were 72.6 Gy_E_, 66 Gy_E_, and 52.8 Gy_E_, respectively. In dose prescriptions to the target volumes and OARs, a relative biological effectiveness (RBE) of 1.1 to Co^60 ^was assumed for the protons. The prescribed doses were normalized to the median dose of the target volumes. Both IMRT and IMPT plans were prepared for each patient to be delivered in 33 fractions with the simultaneous integrated boost technique.

For prepration of IMRT and IMPT plans, the research version of the inverse treatment planning system KonRad (DKFZ, Heidelberg) integrated into the VIRTUOS planning system was used. In IMRT plannings, nine coplanar, equally spaced, 6 MV photon beams were used. For definition of the fluence map, five non-zero intensity levels were chosen. The optimized intensity profile for each beam was then translated into a set of leaf positions for a multileaf collimator, with a resolution of 10 mm at isocenter, simulating a step-and-shoot delivery technique. On average, 132 segments were used for each IMRT plan. In IMPT plans, three coplanar fields (0°, 45°, 315° or 0°, 60°, 300°) were applied. In proton therapy, when target volumes are located in front of critical neural structures such as the spinal cord, an anterior field is usually avoided in order to prevent the distal edge of highly weighted Bragg peaks with uncertain RBEs abutting against the organ. However, for our NPC patients an anterior field was chosen instead of a posterior field to avoid unnecessary exposure of the neural tissues (Cerebellum) behind the nasopharyngeal cavity. For IMPT plannings, we used the 3D spot-scanning technique in which the target volumes were divided into a set of layers with equal radiological depth. For each layer, the treatment planning system generated a discrete beam weight map for regularly spaced pencil beam spots (Bragg peaks) of protons with lateral separation of 5 mm and depth modulation of 3 mm. The initial Full Width at Half Maximum of the proton pencil beams at the patient surface was set to 6 mm. The exact number of the pencil beams were determined by the geometry of the target volumes and the lateral separation of the beam spots. On average, 24,734 spots (range; 15,812 – 39,156) were used for each beam. A simultaneous optimization of the relative weights of the individual proton pencil beams for all three fields was performed by using various pencil beam energies of 160–200 MeV to create the desired dose distributions in the target volumes and OARs.

The inverse optimization process of the plans for both techniques was based on the user-defined dose/dose-volume constraints (Table 1) and relative penalty factors for the target volumes and OARs. For both techniques, all applied dose/dose-volume constraints were soft constraints and they were the same in terms of Gy_E_.

**Table 1:** Dose/volume constraints for OARs in IMRT and IMPT plans

Volume	Dose (Gy_E_)
Spinal cord	*Dmax ≤ *50 Gy_E_
Brainstem	*Dmax ≤ *60 Gy_E_
Temporal lobes	*Dmax ≤ *65 Gy_E_
TM joints	*Dmax *≤ 60 Gy_E_
Optic chiasm & nerve	*Dmax *≤ 54 Gy_E_
Eyes	*Dmax *≤ 25 Gy_E_
Inner ears	*Dmean *< 45 Gy_E_
Mid-external ears	*Dmean *< 40 Gy_E_
Larynx-esophagus	*Dmean *< 30 Gy_E_
Thyroid gland	*Dmean *< 30 Gy_E_
Parotid glands (single gland)	*Dmean *< 26 Gy_E_
Pituitary gland	as low as possible
Oral cavity	as low as possible

Gy_E _= cobalt Gray equivalent, TM = temporomandibular. Dmax is the absolute maximal dose in a single voxel.

In KonRad, each structure classified as target could have a minimum dose, a maximum dose, and an associated penalty factor. Structures classified as OARs could only have maximum doses and associated penalty factors. Optionally, user-defined dose-volume histograms (DVH) could be set for OARs in the program. Furthermore, for overlapping structures (such as GTV-T and temporal lobes in a T4 tumor), the system had to be told which of the structures owned the voxels in the overlap region by assigning the structures priority numbers. Based on the input parameters for target volumes and OARs, KonRad used a single objective iterative optimization algorithm (gradient technique) in order to improve the 3D dose distributions and minimizing the objective functions.

The treatment planning and optimization of IMRT and IMPT was started with cases showing least complex geometry of the target volumes (T1N0M0 and T1N1M0). For target volumes, the minimum and maximum doses were set to be equal to the prescribed dose in order to achieve a maximally homogeneous dose distribution within the target. The critical neural tissues (brainstem, spinal cord, optic apparatus, and temporal lobes) and target volumes were given the highest penalty factors. The initial penalty factors for other OARs were dependent on the importance of their function and their distance from target volumes. For example, the assigned penalty factors for inner ears were higher compared with the middle/external ears. An iterative optimization of the plans was performed by manually adjusting the dose constraints for OARs or penalty factors in a trial-and-error procedure until satisfactory dose distributions in the target volumes and OARs were achieved. No attempt was made to further reduce the dose to OARs below the dose constraints presented in Table 1.

The dose homogeneity and conformity aimed for the target volumes were:

a. dose homogeneity of -5% to +7%.

b. At least 95% of the target volume should receive 95% of the prescribed dose.

c. No more than 5% of the target volume should receive doses above 105% of the prescribed dose.

The actual dose constraints and penalty factors in the final accepted plans from the first two NPC cases were used as starting input parameters for optimization of IMRT and IMPT plans in the subsequent cases. These parameters had to be modified again in a trial-and-error fashion in locoregionally advanced cases in order to comply with the planning goals for the target volumes and/or the tolerance threshold of the critical OARs. In these cases, "optimization only" volumes were also added in order to achieve sharp dose gradients at the edge of the target volumes or to reduce the dose in critical neural tissues such as temporal lobes. In those cases where GTV-T or PTV-TN was extended into a critical neural structure, the latter organ was given a higher overlapping priority than the target. With this approach insufficient dose to some parts of the high dose target volumes (GTV-T and PTV-TN) had to be accepted.

### Plan comparison

The IMRT and IMPT plans were compared using a set of parameters derived from DVHs and dose-volume statistics. Besides *Dmean*, we used *D1 *and *D99*, which were defined as the dose received by 1% and 99% of the target volume, respectively. *V95 *and *V105 *denoted the volumes of the target that were covered with ≥ 95% and ≥ 105% of the prescribed dose, respectively. The *conformity index *(CI) was defined as the ratio between the V95 of the body and the V95 of the target. The inhomogeneity coefficient (IC) was defined as (*Dmax *- *Dmin)/Dmin*. For PTV-TN and PTV-N, all parameters were calculated for inclusive volumes of the targets due to the limitations of the VIRTUOS planning system in calculating exclusive volumes. The term "inclusive volume" means that the volumes of GTV-T and PTV-TN were included in the PTV-TN and PTV-N, respectively, when calculating and extracting the dose-volume data for the latter targets. Ideally, when a target encloses another one, dose-volume data for the first target should be presented by excluding the dose contributions from the enclosed target when this receives a dose other than the enclosing target.

For comparison of OARs, we used *Dmax *and *Dmean *for organs with mainly parallel structures and *Dmax *for those with mainly serial structures. *Dmax *for OARs was defined as the absolute maximal dose in a single voxel.

### Statistics

Statistical analysis was performed using Wilcoxon signed ranks tests applying SPSS 12.0.1 software for windows. A two-tailed *p*-value of < 0.05 was accepted as significant.

## Results

### Targets

**Figure 1:**
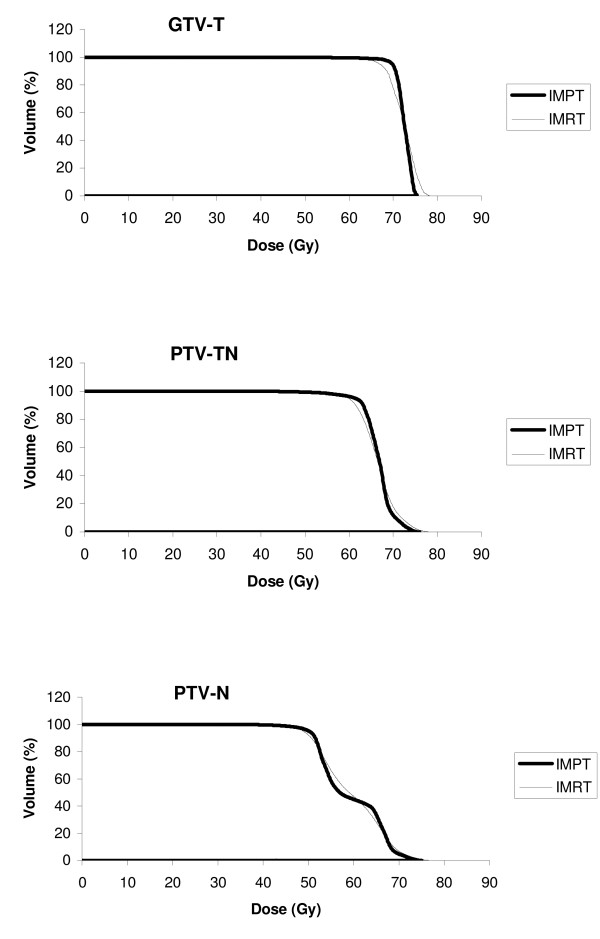
Mean DVH curves of 8 NPC patients for target volumes comparing IMPT with IMRT.

Table 2. presents the averaged dosimetric parameters for all three target volumes, comparing IMPT with IMRT plans. There were no significant differences in *Dmean *or *D99 *for any target volume, except for the averaged *D99 *of PTV-TN, which was significantly (2.8 Gy_E_) lower in IMPT plans. The averaged *Dmean *for PTV-N (59 Gy_E_) in both techniques was higher than the prescribed dose (52.8 Gy_E_), which partly was a result of dose calculation for the inclusive volume (including PTV-TN and GTV-T) of this target. For all target volumes, *D1 *was always lower in IMPT plans by an average value of 1.3 Gy_E_. Similarly, mean *V105 *values were lower in IMPT than IMRT plans for all target volumes, although the difference for GTV-T was not statistically significant. The averaged and individual values for *V95 *were almost always better in IMPT than in IMRT plans, reflecting better tumor coverage. This resulted in an increase of the averaged *V95 *by 3.4% for PTV-N, 5.6% for PTV-TN, and 4.6% for GTV-T in IMPT plans. Figure 1. shows the mean DVHs for target volumes obtained for all eight NPC patients comparing IMPT with IMRT plans.

**Table 2:** Mean dose-volume data and standard deviations for 8 NPC patients comparing IMPT with IMRT

Target	Parameter	IMPT		IMRT		P value
		Mean +/- 1SD		Mean +/- 1SD		
GTV-T	D99	66.6	6.1	64.9	4.2	0.195
	D1	74.8	1.1	76.1	0.8	0.008
	*D. mean*	72.4	0.4	72.2	0.4	0.148
	SD	1.4	1.07	2.2	0.9	0.008
	V95	97.6%	3%	93%	6%	0.016
	V105	0.5%	0.9%	1.6%	2%	0.063
	CI	2.36	1.07	4.69	3.59	0.008
	IC	0.11	0.1	0.17	0.09	0.016
						
PTV-TN	D99	52.5	4.2	55.3	2.2	0.039
	D1	73.4	0.6	74.7	0.7	0.008
	*D. mean*	66.5	0.4	66.5	0.2	0.844
	SD	3.4	0.7	3.8	0.54	0.188
	V95	93.3%	2%	87.7%	3%	0.008
	V105	12.2%	5%	19.7%	4%	0.008
	CI	1.02	0.07	1.12	0.12	0.016
	IC	0.32	0.07	0.32	0.05	0.844
						
PTV-N	D99	45	2.3	44.7	0.9	0.945
	D1	72.5	1.2	73.6	1.2	0.008
	*D. mean*	59.3	2.7	59.5	2.3	0.742
	SD	6.7	0.84	7	0.92	0.219
	V95	95.4%	2%	92.0%	2%	0.016
	V105	52.9%	17.9%	60.5%	13.8%	0.047
	CI	1.11	0.06	1.32	0.12	0.016
	IC	0.55	0.07	0.6	0.05	0.383

All parameters are shown for the inclusive volumes of PTV-TN and PTV-N. SD = standard deviation, CI = conformity index, IC = inhomogeneity coefficient. Values for D99, D1, D. mean and SD are in cobalt Gray equivalent (Gy_E_).

**Figure 2:**
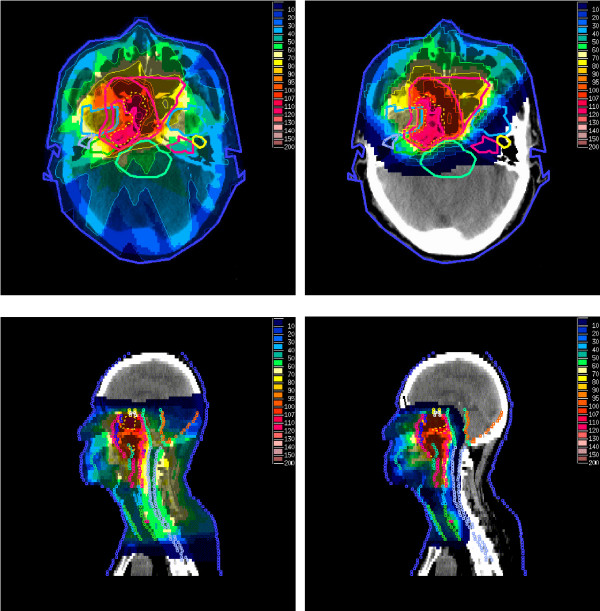
Comparison of dose distributions between IMPT (right) and IMRT (left) plans in T4N1M0 NPC in axial (above) and sagittal (below) views. Dotted lines denote 95% of the prescribed dose to GTV-T.

**Figure 3:**
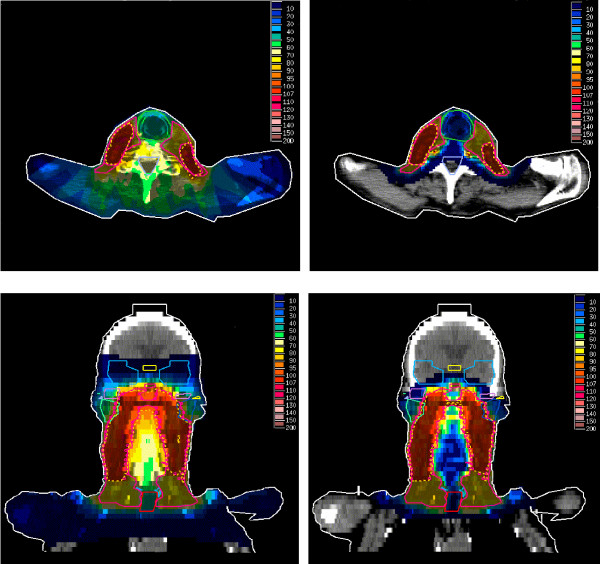
Comparison of dose distributions between IMPT (right) and IMRT (left) plans in T2N3M0 NPC in axial (above) and coronal (below) views. Dotted lines denote 95% of the prescribed dose to PTV-TN.

The individual and mean values for CI were always better in the IMPT plans for all targets except in one case (T3N2M0) for PTV-TN, where they were almost equal for both plans (1.07). In both techniques, the best CI values were obtained for PTV-TN volumes (average value,1.02 vs. 1.12). The corresponding values were much higher for GTV-T (average value; 2.36 vs. 4.68) reflecting the difficulty both treatment techniques had in avoiding small islands of 95% isodose in the rest of the treatment/target volumes. The evaluation of dose inhomogeneity measured by IC showed significant superiority of IMPT for GTV-T (mean value: 0.11 vs. 0.17). There was no significant difference between the two techniques for other target volumes. However, the latter result could be misleading since inclusive volumes of PTV-TN and PTV-N were used for DVH calculations. Figure 2 and 3. present the dose distribution in different planes for two NPC cases.

### Organs at risk

**Figure 4:**
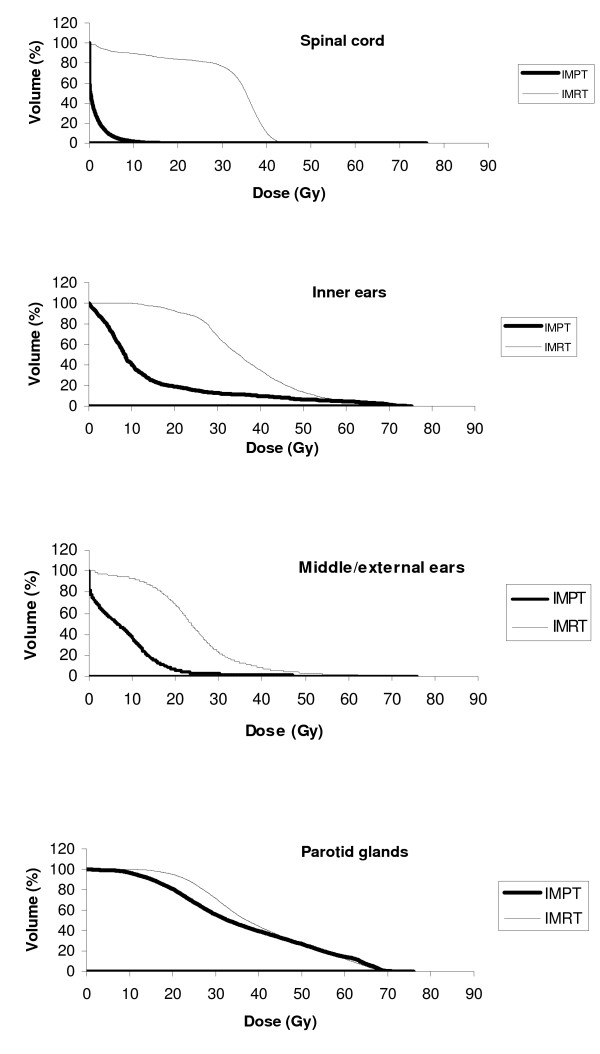
Mean DVHs for OARs comparing IMPT with IMRT.

Table 3. compares the averaged dose parameters for OARs between the IMPT and IMRT plans. In brief, the averaged *Dmax/Dmean *for most of OARs was significantly lower in the IMPT plans. Exceptions were the values for *Dmax *of the brainstem, TM joints, oral cavity, pituitary gland, and the skin and for *Dmean *of the pituitary gland. For locally advanced tumors, IMPT plans had as much difficulty as IMRT plans in lowering the *Dmax *to OARs located in the vicinity of the GTV-T covered by the high isodoses. In some of these cases, individual *Dmax *values (measured in single voxel volumes) for the inner and middle/external ears and TM joints were in fact somewhat higher in IMPT plans. The dosimetric superiority of the IMPT plans was reflected in the *Dmean *of OARs such as the auditory apparatus, temporal lobes, TM joints, larynx/esophagus, and thyroid gland, where the averaged values were one-third to one-half of the corresponding values in the IMRT plans. For the spinal cord, the averaged *Dmax *was halved by IMPT plans. The averaged *Dmax *and *Dmean *for cerebellum and posterior brain tissue up to the level of clinoids were also significantly lower in IMPT plans (35 Gy_E _and 0.5 Gy_E_) compared to IMRT plans (57.2 Gy_E _and 18.8 Gy_E_) even though these structures were not considered initially in the optimization process. The averaged *Dmax *for the skin was almost equal for both modalities (65.7 Gy_E _vs. 66.8 Gy_E_) but the averaged *Dmean *was significantly lower in IMPT plans (5.7 Gy_E _vs. 9.6 Gy_E_). Figure 4. shows mean DVHs of some OARs for the two modalities.

**Table 3:** Mean dose parameters in Gy_E _for OARs in 8 NPC patients planned with IMPT and IMRT

Organ/	IMPT	IMRT	IMPT/IMRT	P value
Parameter	Mean (Range)	Mean (Range)	Mean dose ratio	
Optic chiasm				
D. max	16.1 (0.0 – 52.3)	23.8 (4.8 – 56.8)	0.67	0.008
Spinal cord				
D. max	16.7 (10.1 – 28.7)	46.0 (44.0 – 50.0)	0.36	0.008
Brainstem				
D. max	47.3 (14.6 – 60.5)	58.7 (53.6 – 64.2)	0.80	0.055
Temp. lobe				
D. max	57.2 (33.0 – 67.8)	61.8 (53.5 – 67.7)	0.92	0.021
D. mean	5.4 (0.5 – 12.3)	13.4 (8.4 – 20.3)	0.40	< 0.0001
Inner ear				
D. max	36.3 (8.0 – 76.1)	51.8 (32.8 – 73.0)	0.70	0.009
D. mean	13.1 (1.1 – 43.9)	36.4 (21.8 – 53.0)	0.36	< 0.0001
Mid/ext ear				
D. max	37.4 (8.0 – 70.4)	49.2 (33.0 – 68.2)	0.76	0.039
D. mean	8.1 (0.3 – 13.5)	24.5 (19.4 – 32.8)	0.33	< 0.0001
TM joint				
D. max	54.9 (37.0 – 73.0)	60.5 (45.9 – 71.4)	0.90	0.252
D. mean	17.4 (7.3 – 28.9)	38.8 (27.9 – 54.6)	0.44	< 0.0001
Larynx/esophgus				
D. max	52.8 (46.6 – 64.4)	57.9 (52.3 – 64.1)	0.91	0.039
D. mean	14.3 (8.6 – 18.5)	30.6 (24.3 – 35.3)	0.46	0.008
Oral cavity				
D. max	70.2 (68.6 – 73.9)	72.1 (70.4 – 74.4)	0.97	0.055
D. mean	38.1 (33.9 – 43.7)	44.0 (40.5 – 46.7)	0.86	0.016
Pituitary gl.				
D. max	44.9 (3.2 – 73.0)	53.5 (20.6 – 73.8)	0.84	0.055
D. mean	34.8 (1.1 – 71.0)	42.2 (11.6 – 65.9)	0.82	0.148
Thyroid gl.				
D. max	51.5 (42.4 – 64.2)	57.5 (52.9 – 64.9)	0.89	0.008
D. mean	19.8 (12.5 – 24.4)	38.2 (33.7 – 45.4)	0.52	0.008
Parotid gl.				
D. mean	36.3 (23.6 – 44.3)	40.0 (26.0 – 49.0)	0.91	0.011

Gy_E _= cobalt Gray equivalent, TM = temporomandibular, Mid/ext = middle/external. D. max is the absolute maximal dose in a single voxel.

### Non-specific normal tissue

**Figure 5:**
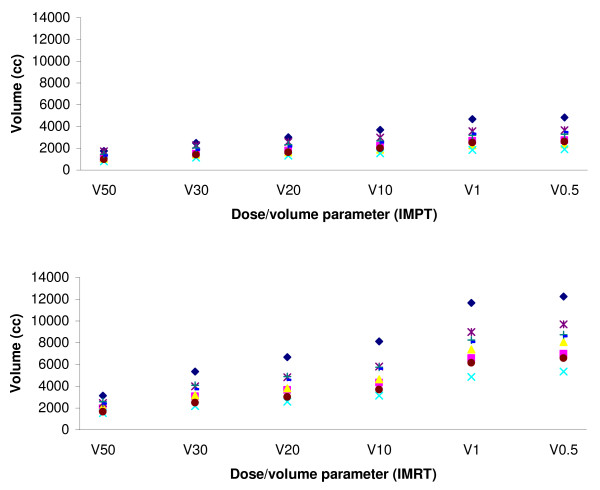
Volumes of low-medium isodoses (0.5%–50%) in IMPT and IMRT for 8 NPC patients.

The dose to non-specific normal tissues was measured by calculating V50, V30, V20, V10, V1, and V0.5 of the body, corresponding to the volumes of the 33 Gy_E_, 19.8 Gy_E_, 13.2 Gy_E_, 6.6 Gy_E_, 0.66 Gy_E_, and 0.33 Gy_E _isodoses. The obtained results for each technique and for all eight patients are shown in Figure 5. On average, for each of the above isodoses, IMRT plans resulted in increments that were 1.78, 1.99, 2.06, 2.11, 2.57 and 2.66-fold greater than the IMPT plans.

## Discussion

In terms of RT treatment planning, NPC is one of the most difficult diagnoses in the head and neck region due to the complex geometry of the tumor and the several critical and functional structures surrounding the target. The clinical advantages of IMRT in NPC have been demonstrated through non-randomized clinical studies [6,7,17], which show improved 2–4 year local/locoregional control rates of 88–98%, no grade III xerostomia, and a reduced rate of grade III–IV hearing loss to 7–15%. However, one problem with the published clinical data on IMRT of NPC patients is the small sample size and short follow-up period in evaluation of patterns of tumor failure and late normal tissue reactions, including the risk of RT-induced second malignancies. Furthermore, the high rate of tumor control in such studies could be confounded by the effects of accelerated RT or combined modality treatment using chemotherapy [18].

Recently, much effort has been dedicated to evaluating proton therapy, especially IMPT, for different tumor sites including the head and neck region [19-24]. Most of the published data from the comparative planning studies suggest equivalent levels of target conformation with both IMRT and IMPT techniques. The superiority of IMPT is attributed mostly to lower integral doses in OARs and non-target volumes and to the possibility of dose escalation to the tumor [15,20,22,25]. These observations are partially supported by the results of the current study. In our IMPT plans, the averaged *D99 and D. mean *did not differ significantly from those for IMRT plans, except for the averaged *D99 *of PTV-TN, which was, interestingly, 2.8 Gy_E _lower in IMPT plans probably as the result of the limited number of the fields (three) used in preparation of IMPT plans. In the case of GTV-T, however, averaged values for *D1*, *V95*, CI and IC were all significantly improved by IMPT, even though the magnitude of the absolute differences was more appreciable for *V95 *(4.6%) and CI (2.33). Technically, tumor coverage was more compromised in IMRT plans when targets were closely surrounded by several critical OARs with maximum dose-constraints below the prescribed dose to the target. The typical cases were intracranially extended T4 tumors surrounded by temporal lobes at both sides, the optic apparatus in front and brainstem at back. This problem was less pronounced in IMPT plans in which 3D modulation of the fluences of the fields gave more degree of freedom in the treatment planning.

It is possible that we could have improved the conformity of the IMRT plans further by using higher intensity levels than five when preparing the plans. However, the expected gain would be slight as it has been suggested by Longobardi et al. [26]. In their planning study of seven patients with head and neck cancer in which IMRT with step and shoot technique and various intensity levels of 5, 10, and 20 were included, doses of 54 Gy and 64.8–70.2 Gy were prescribed for PTV1 and PTV2 (boost volume), respectively. The authors reported V95 values of 94%, 95.4%, and 95.9% for the three intensity levels for PTV1. Corresponding values for PTV2 were 86.4%, 87.9%, and 87.9% respectively.

We must point out that the optimization of the beam intensity profiles in our IMRT and IMPT plannings were based on a single objective inverse treatment planning algorithm. One of the inherent limitations of this approach is the fact that choice of the input parameters such as interstructural penalty factors in achieving an optimal plan is a trial-and-error procedure and it is based on the empirical knowledge of the planner. Besides being time consuming depending on the number of optimization cycles, single objective inverse treatment planning does not explore the optimization search space to its full extent and in the end, it is not certain whether an accepted plan is the best clinically achievable solution. Recently, multicriteria optimization systems for inverse treatment planning in RT has been introduced [27]. With this approach, a large collection of clinically relevant treatment plans based on the pareto-optimal solution is precomputed and as the result the trial-and-error approach in the optimization step is avoided. Through navigation in a multidimensional plan space, the user can identify the advantageous trade-offs in competing plans and select the plan that achieves the optimal clinical objectives. The incorporation of multicriteria optimization in comparative inverse treatment planning studies in RT may provide more accurate and fare data in ranking of various RT techniques.

In this study, no attempt was made to escalate the dose beyond 72.6 Gy_E _for any T stage. The reasons for this decision were, firstly, that reported local recurrences in IMRT studies of NPC patients who received radiobiologically equivalent doses, as in the current study, were attributed only to more advanced (T3–T4) tumors [6,7,17] which did not warrant further dose-escalation for T1–T2 tumors. Secondly, in T3–T4 tumors, *Dmax *calculated in a single voxel for the temporal lobes was already in the order of 67 Gy_E _in both IMRT and IMPT plans, reflecting the limitations of both techniques when it comes to reducing maximum doses in OARs in the vicinity of the target. Dose-escalation to partial volumes of the primary tumor (boost within the boost volume), especially for the hypoxic regions which can be identified by functional imaging, is an option to be explored with both techniques. Boost within the boost principle might alternatively be applied to residual tumor mass after certain delivered dose.

Locoregional recurrences of NPC without distant failure are believed to be potentially curable [28]. However, in salvage therapy of local recurrences, the success of intracavitary brachytherapy or nasopharyngectomy is restricted to the limited target volumes and application of external RT with photons is associated with severe side effects [28]. Reduction of the integral doses to several OARs, including the spinal cord in primary treatment of NPC with IMPT could leave more room for optimal target treatment and sparing of critical structures in re-irradiation situations. Furthermore, despite the promising role of IMRT in local/locoregional tumor control of NPC, systemic failure still occurs in many patients, yielding 4-year distant metastases-free rates as low as 66% [6]. Accordingly, combined chemoradiotherapy is recommended for locoregionally advanced NPC [29], which necessitates implementation of new RT techniques whose side effects are less potentiated in a multimodal treatment setting.

Xerostomia is one of the most common RT-related side effects in NPC patients. Although our IMPT plans could significantly reduce the averaged *Dmean *of parotids (36.3 vs. 40.0 Gy_E_), the obtained mean values for both techniques were higher than those reported by other proton (25.8 – 33 Gy_E_) and IMRT (33–35.2 Gy_E_) studies [6,7,10,11,17]. Different results in parotid sparing may relate to variations in target volume definition, especially for elective neck treatment. In the current study, deep parotid lobes were always included in PTV-Ns which inevitably lead to relatively higher *Dmean *values in the majority of the glands.

Therapy-induced hearing deterioration is another frequent complaint in NPC patients [30]. It has been suggested that mean doses of 45–48 Gy_E _(32 Gy_E _in children) to inner ears can lead to significant loss of hearing [31-33], which supports the benefits of our IMRT and IMPT plans in lowering the averaged *Dmean *of inner ears (36.1 Gy_E _vs. 13.1 Gy_E_). However, only IMPT could keep the *Dmean *low enough for all patients and ears, a finding, which is especially beneficial in locoregionally advanced NPC for which cisplatin-based chemotherapy is often used. For middle and external ears, the averaged *Dmean *was reduced by a factor of 3 by IMPT, which is especially appreciated considering the report of a 30% incidence rate of otitis media in 20 NPC patients who had received an IMRT dose of 72 Gy_E _(2.4 Gy_E_/fraction) [17].

The rate of moderate to severe trismus in NPC patients treated by traditional RT techniques can be as high as 30% [4]. In the case of the TM joints, 50 Gy_E _is reported as the critical level for induction of apparent trismus even though, functional joint impairments have been reported with doses as low as 15 Gy_E _[34], supporting the benefits of IMPT as shown in this study.

The carcinogenic effect of ionizing radiation has been a matter of debate, especially for low-dose irradiated volumes. In a long-term follow-up of the 28,008 Swedish infants irradiated for skin hemangioma, Karlsson et al. [35] found a strong linear dose-response relationship between absorbed dose in the brain and subsequent risk of developing an intracranial tumour. In their study, the mean intracranial doses in the total cohort and in the individuals developing tumors were estimated as 0.07 Gy_E _and 0.31 Gy_E_, respectively. In another survey of 53 patients with second malignancies after irradiation to different anatomical locations, most of the tumors were found in the volume receiving ≤ 6 Gy_E _[36]. In our study, the averaged volumes of 0.33 Gy_E _to 13.2 Gy_E _were more than doubled by IMRT, which is of great concern especially in children. The pediatric patients account for between 1–18% of all NPC cases [37] and they are often treated using the same RT guidelines established for adults. In a survey of 33 cases of childhood NPC treated with older RT techniques to a median dose of 60 Gy_E_, the 10-year actuarial rate of severe complications was reported to be as high as 24% and two cases of second malignancies in salivary glands were observed after median latency of eight years [38]. Among other side effects, iatrogenic endocrine insufficiency is often observed in children who received RT to the head and neck region due to the inclusion of the hypothalamic-pituitary axis and thyroid gland in the radiation fields. Our IMPT plans reduced the averaged *Dmean *of the thyroid gland by a factor of two. However, for the pituitary gland, neither technique was able to reduce the *Dmean *values sufficiently for locally advanced tumors. In general, it seems that for pediatric NPC patients, IMPT combined with the spot-scanning technique is the best RT option for sparing OARs and minimizing the risk of second malignancies. Since collimation, compensation, and scattering are not applied in spot-scanning of protons, the risk of neutron contamination and consequent malignancy induction is also reduced [39].

It is imperative to acknowledge some pitfalls of using protons in NPC patients as the dose to the skin surface, especially in cases with large and bilateral lymph node metastases. In our study, the averaged *Dmax *for skin was equal for IMPT and IMRT techniques (66 Gy_E_) and averaged *Dmean *was low (< 10 in Gy_E_) in both techniques. Although treatment planning systems can fall short in accurate dose calculations in the skin, our results suggest that acute skin toxicities should not be any more severe in IMPT than IMRT of NPC patients. Severe skin reactions are probably more of a problem with passive scattering single field proton therapies.

For the IMPT plannings, we used a standard proton pencil beam algorithm with x-ray CT-based path length scaling. Possible errors in conversion from CT numbers to stopping power of proton pencil beams (range errors) especially in the presence of complex air-bone interfaces along the proton beam path in the nasopharyngeal region could be a source for uncertainties in the Bragg peak positioning. Application of a Monte Carlo based treatment planning algorithm could have modelled the complex geometries involved in IMPT of NPC patients more accurately and thereby it could have reduced the probability of range errors [40]. For complex and highly accurate RT delivery techniques such as IMRT and IMPT, knowledge of the exact location of the targets and OARs prior to each RT treatment is also essential. This is especially important in proton therapy since changes in the tissue heterogeneities due to shrinkage of the tumor mass, weight loss or development of sinusitis as in RT of NPC patients may all cause range-shifting of the proton beams considerably. These together with deviations of the target position in daily setup of the patients may induce significant real time cold and hot spots within and outside the target. Such problems can be minimized by application of Image-guided proton therapy (IGPT) during the course of the treatment. Based on the acquired information, both correction for the daily deviations of the target position and modifications of the treatment plans in case of significant changes in the patient geometry become possible. IGPT can be accomplished by using in-room CTs like linac attached x-ray systems, CT scanner on rails, or functional treatment verification imaging based on the reconstruction of positron emission tomography signals derived from the nuclear interactions in the trajectory of proton beams [41].

Another issue to consider in IMPT plannings is the uncertain RBE values at the distal edge of spread-out Bragg Peaks abutting neural structures. While expected variations of RBE values for locally limited NPC with longer distance to neural tissues will have no practical impact in the primary treatment of the lesions, there are grounds for concern for tumors growing in the vicinity of these structures. In the current study, more strict dose constraints were chosen for critical OARs, partially based on the small uncertainties in the range of the protons and distal-edge RBE effects in IMPT plans. For instance, we defined *Dmax *for both surface and center of the brainstem and optic apparatus as 60 Gy_E _and 54 Gy_E_, respectively. Although clinically relevant for both techniques, these dose constraints were lower than what is currently used by some proton therapy centers (<64 Gy_E _as surface dose for brainstem and <60 Gy_E _for the optic apparatus) [42]. A more attractive approach would be to implement methods to estimating 3D RBE variations within target and non-target volumes and to consider them during the optimisazion of the IMPT plans, as presented by Wilkens et al. [43] for prostate cancer. Although their method was based on experimental and in vitro data, it seemed to be a promising step for better clinical evaluation and optimization of IMPT plans.

In the end, we do acknowledge that until we have powerful models to predict the accurate positioning of spread-out Bragg peaks of the protons and their distal-edge RBE values before and during course of IMPT, the practical use of our IMPT planning approach especially with a beam direction at zero degree may be limited. Furthermore, the delivery of IMPT in NPC patients with the current approach demands not only scanning beams but also a rotating gantry in order to target the tumor with the most optimal beam directions.

## Conclusion

In treatment of NPC, three-field IMPT is dosimetrically superior to nine-field IMRT concerning tumour coverage and conformity. However, the major gain with IMPT is the reduction in the integral doses to several OARs and non-specific normal tissues. The actual benefits and practicality of this technique for NPC patients can only be verified through carefully designed clinical trials when IMPT is available on wider clinical scale.

## Competing interests

The author(s) declare that they have no competing interests.

## Authors' contributions

ZTK and MWM designed the study, performed the treatment plans, interpreted the results of the study, and oversaw the project completion. SN and JJW participated in preparing of the treatment plans and data acquisition. ZTK performed the statistical analysis and drafted the manuscript. All authors contributed to the scientific setup of the study and revised the manuscript critically and they have approved the final version of the manuscript.
